# Elective nodal irradiation with simultaneous integrated boost stereotactic body radiotherapy for pancreatic cancer: Analyses of planning feasibility and geometrically driven DVH prediction model

**DOI:** 10.1002/acm2.12528

**Published:** 2019-01-13

**Authors:** Akira Nakamura, Hugh A. Prichard, Jennifer Y. Wo, John A. Wolfgang, Theodore S. Hong

**Affiliations:** ^1^ Department of Radiation Oncology Massachusetts General Hospital Harvard Medical School Boston MA USA

**Keywords:** pancreatic cancer, planning study, prophylactic irradiation, SBRT

## Abstract

**Purpose:**

We evaluate the feasibility of the elective nodal irradiation strategy in stereotactic body radiotherapy (SBRT) for pancreatic cancer.

**Methods:**

Three simultaneous integrated boost (SIB)‐SBRT plans (Boost1, Boost2, and Boost3) were retrospectively generated for each of 20 different patients. Boost1 delivered 33 and 25 Gy to PTV1 and PTV2, respectively. Boost2 delivered 40, 33, and 25 Gy to boostCTV, PTV1, and PTV2, respectively. Boost3 delivered 33 and 25 Gy to PTV1 and PTV3, respectively. PTV1 covered the initial standard SBRT plan (InitPlan) gross tumor volume (GTV). PTV2 covered CTVgeom which was created by a 10‐mm expansion (15 mm posterior) of GTV. PTV3 covered CTVprop which included elective nodal regions. The boostCTV included GTV as well as involved vasculature. The planning feasibility in each scenario and dose–volume histograms (DVHs) were analyzed and compared with the InitPlan (delivered 33 Gy only to PTV1) by paired *t*‐test. Next, a novel DVH prediction model was developed and its performance was evaluated according to the prediction accuracy (AC) of planning violations. Then, the model was used to simulate the impacts of GTV‐to‐organs at risk (OAR) distance and gastrointestinal (GI) OAR volume variations on planning feasibility.

**Results:**

Significant dose increases were observed in GI‐OARs in SIB‐SBRT plans when compared with InitPlan. All dose constraints were met in 63% of cases in InitPlan, Boost1, and Boost2, whereas Boost3 developed DVH violations in all cases. Utilizing previous patient anatomy, the novel DVH prediction model achieved a high AC in the prediction of violations for GI‐OARs; the positive predictive value, negative predictive value, and AC were 66%, 90%, and 84%, respectively. Experiments with the model demonstrated that the larger proximity volume of GI‐OAR at the shorter distance substantially impacted on planning violations.

**Conclusions:**

SIB‐SBRT plan with geometrically defined prophylactic areas can be dosimetrically feasible, but including all nodal areas with 25 Gy in five fractions appears to be unrealistic.

## 1 INTRODUCTION

Stereotactic body radiotherapy (SBRT) is increasingly being used for unresectable, locally advanced pancreatic cancer (LAPC) as well as in the neoadjuvant setting for borderline resectable diseases.^1^ SBRT delivers larger radiation doses in three to five fractions with more conformable dose distribution than conventional chemoradiation therapy (CRT).^2^ The small target volumes of pancreatic SBRT without covering any prophylactic nodal areas allow steeper dose gradients and a better sparing of abdominal organs at risk (OAR),^3^, ^4^, ^5^ thus potentially limiting the toxicity.

Data from multiple studies of neoadjuvant CRT highlight the prognostic importance of the margin status and residual nodal involvement in resected pancreatic cancer.^6^, ^7^, ^8^, ^9^, ^10^ To date, limited data have been available on these factors from patients who received SBRT and resection.^11^, ^12^, ^13^ Polistina et al.^11^ reported that two patients underwent resection following neoadjuvant chemotherapy and SBRT, and both had at least two positive peripancreatic lymph nodes despite the negative margin and good local responses. Mellon et al.^13^ reported that fewer positive lymph nodes were observed in the neoadjuvant SBRT group in comparison with patients who received upfront surgery (42.6% vs 60.6%). These results suggest that the neoadjuvant short‐course SBRT is a viable approach, although treatment effects on micrometastases to regional lymphatics were still unsatisfactory. Given the prognostic importance of resection margin status as well as lymph node positivity in pancreatic cancer, the prophylactic irradiation may improve outcomes of pancreatic SBRT, if it can be applied without increasing severe toxicities.

Previously, we conducted phase I studies of preoperative short‐course radiotherapy,^14^, ^15^ which included prophylactic nodal area irradiation, and we experienced unexpected intraoperative complications from photon therapy although its prescription dose was relatively modest (25 Gy in five fractions). Dosimetric analyses found that, compared with proton therapy, larger volumes of gastrointestinal (GI) OARs had been irradiated with low doses of photon therapy,^15^, ^16^ which were thought to lead to intraoperative complications.^17^ With growing evidences for the pancreatic SBRT dosimetry^3^, ^4^, ^5^ and recent clinical studies on the dose–toxicity relationship,^18^, ^19^, ^20^ strict dose constraints on GI‐OARs have been accepted.^21^, ^22^ Achieving these constraints would be necessary to establish a safe SBRT, even with prophylactic area irradiation. In the meantime, impacts of patient anatomy on the planning feasibility have been reported.^3^, ^4^ Important questions for SBRT prophylactic area irradiation include how geometrical information relates to the planning feasibility as well as to what extent regions can be safely treated. Therefore, a fundamental study to evaluate its feasibility is needed before the introduction of this approach to the clinical setting.

**Figure 1:**
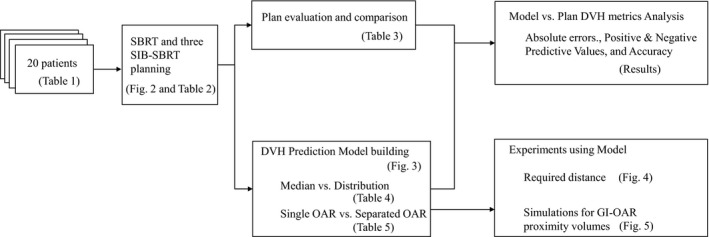
Flow diagram of study design.

The purpose of this study was twofold: to retrospectively generate the treatment plans of standard tumor‐only SBRT and of an alternative dose painting SBRT strategy to cover relevant nodal volume, vascular involvement, or prophylactic areas. Then, we evaluated the ratio of successful treatment planning in each scenario. Next, we developed a new geometry‐driven dose–volume histogram (DVH) prediction model to evaluate the geometry–dosimetry relationship and elucidate what factors were crucial for the failure planning to achieve dose constraints in SIB‐SBRT for patients with pancreatic cancer. Figure 1 outlines a flow diagram of the dataset and the SBRT planning, DVH prediction models, and experiments introduced in the following sections.

## 2 MATERIALS AND METHODS

### 2.A Patient and treatment planning

#### 2.A.1 Patient selection and target definition

This was a single‐institution retrospective planning study, approved by the Institutional Review Board. A total of 20 patients with pancreatic cancer who received radiotherapy were selected (Table 1). The planning computed tomography (CT) images and digital image communications in medicine‐radiation therapy (DICOM‐RT) structure set were deidentified and exported to the treatment planning system. All simulation CTs were performed with a contrast‐enhanced agent without oral contrast. Patients were in the prone position with both arms raised overhead.

**Table 1:** Patient characteristics and tumor size

Pt.	Gender	Age	Tumor	GTV	CTVgeom	CTVprop	PTV1	PTV2	PTV3
1	M	69	Body	5	47	349	10	68	428
2	M	59	Head	13	70	259	25	98	336
3	M	73	Tail	29	144	91	57	196	143
4	F	38	Head	101	303	227	136	431	315
5	F	73	Head	89	278	271	120	377	363
6	M	60	Head	61	215	441	88	286	553
7	M	51	Head	33	122	424	47	166	545
8	M	77	Head	56	210	401	83	284	469
9	M	52	Body	70	212	427	107	274	544
10	F	80	Head	26	111	221	39	154	290
11	F	77	Head	8	58	140	17	83	195
12	M	81	Head	39	188	368	73	254	473
13	M	83	Head	9	55	304	18	78	404
14	F	73	Head	57	218	339	95	282	452
15	F	71	Head	4	40	75	11	59	112
16	M	79	Head	35	167	259	66	217	346
17	F	56	Head	16	94	349	33	129	462
18	M	64	Head	24	117	235	45	157	395
19	F	71	Body	38	178	336	71	237	436
20	F	48	Head	16	101	241	34	140	327
Mean		67		36	146	288	59	199	379
Median		71		31	133	288	52	181	400
Min		38		4	40	75	10	59	112
Max		83		101	303	441	136	431	553

Structure volume are shown in mL.

Pt. = patient number; M = male; F = female; GTV = gross tumor volume; CTV = clinical target volume; CTVgeom = geometrical CTV; CTVprop = prophylactic CTV; PTV = planning target volume.

**Figure 2:**
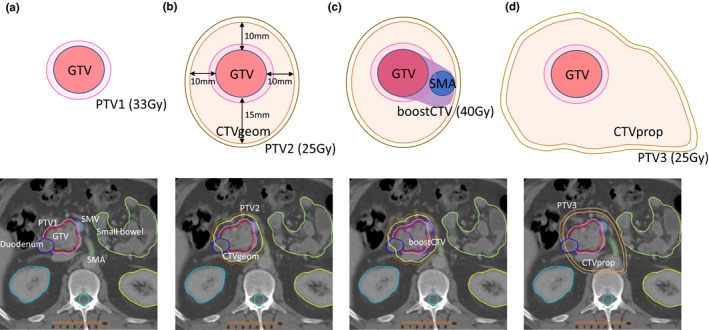
Definitions of target volumes for SBRT and SIB‐SBRT plans. Red = gross tumor volume (GTV), yellow = geometrically defined clinical target volume (CTVgeom) which are created by expanding 10–15 mm from GTV, orange = clinical defined prophylactic clinical target volume (CTVprop), and purple = boost CTV for GTV, as well as the involvement of vasculature. Pink = planning target volume 1 (PTV1) which is created by expanding 3 mm from GTV, brown = PTV2 which is created by expanding 3 mm from CTVgeom, and light brown = PTV3 which is created by expanding 3 mm from CTVprop. PTV1, PTV2, PTV3, and boostCTV receive 33, 25, 25, and 40 Gy, respectively. Both b and c include the same CTVgeom while the boostCTV is also created in c. These target volumes are used for (a) InitPlan, (b) Boost1, (c) Boost2, and (d) Boost3.

The gross tumor volume (GTV) was delineated by treating radiation oncologists, and OAR including the stomach, duodenum, bowel, kidneys, liver, and spinal cord were delineated and used for the plan optimization. Three clinical target volumes (CTVs) were defined; first, a geometrical CTV (CTVgeom) was created with a 10‐mm isotropic expansion (15 mm posterior) of the GTV. Second, boostCTV was defined as the target volume of GTV in continuity with the involved vasculature, as well as the retroperitoneum posterior to the SMV/SMA or celiac axis. In addition, a part of boostCTV which was within 1 cm from the surface of the GI‐OARs was omitted. Third, prophylactic CTV (CTVprop) was defined as GTV plus a prophylactic lymph node area, namely peripancreas, retropancreas, portahepatis, and para‐aorta, according to the tumor location. These CTVs were selected because these regions are clinically relevant for the micrometastasis to regional lymphatics or the microscopic extension from primary tumor.^6^, ^7^, ^8^, ^9^, ^10^ Also, recent practical guidelines recommend CTV margin of 5–15 mm expansion from GTV.^23^ In support of this, we previously reported the tumor size discrepancy between the CT and the pathological sample and suggested a GTV‐to‐CTV margin formula based on a diameter of tumor.^24^ With these facts, we performed preliminary experimental planning using several margin sizes and determined the margins for CTVgeom. Figure 2 illustrates these definitions of target structures.

Three definitions of planning target volume (PTV) were created; PTV1, PTV2, and PTV3 were defined as a 3‐mm isotropic expansion of the GTV, CTVgeom, and CTVprop, respectively. Of note, the overlapping volumes between PTVs and critical normal structures, including the stomach, duodenum, and small bowels, were subtracted from PTV1, PTV2, and PTV3. Table 2 summarizes these definitions and differences of the structures in the plans described in the following section.

**Table 2:** Definitions of target structures in SBRT and SIB‐SBRT plans

	Structure CTVgeom	boostCTV	CTVprop	PTV1^b^	PTV2	PTV3
Definitions	GTV + 10‐mm (15‐mm posterior) expansion	GTV + involved vasculature + retroperitoneum posterior to SMV/SMA or celiac axis	GTV + elective nodal regions (peripancreas, retropancreas, porta‐hepatis, para‐aorta)	GTV + 3‐mm	CTVgeom + 3‐mm	CTVprop+ 3‐mm
Prescription a						
InitPlan				**33 Gy**		
Boost1				**33 Gy**	25 Gy	
Boost2		**40 Gy**		33 Gy	25 Gy	
Boost3				**33 Gy**		25 Gy

Abbreviations: GTV, gross tumor volume; CTV, clinical target volume; CTVgeom, geometrical CTV; CTVprop, prophylactic CTV; PTVx, planning target volume 1, 2, or 3.

a Prescription doses in SBRT plan (InitPlan) or SIB‐SBRT plans (Boost1, Boost2, or Boost3) are shown. Each prescription dose is specified to D95 of the target structure(s), and the most prioritized structure in each plan was shown as bold underline.

b The overlapping volumes between PTVx and critical normal structures were subtracted from PTVx.

#### 2.A.2 Treatment planning and Intensity‐modulated radiation therapy (IMRT) optimization

The IMRT treatment planning was performed on the RayStation (version 4.034, RaySearch, Stockholm, Sweden). Seven to nine coplanar or noncoplanar 6‐MV photon beams were used. Four concepts of SBRT plans for each patient were generated; for the standard SBRT plan (InitPlan), the prescription dose of 33 Gy was specified to D95 (the dose that covers 95% of the structure) of PTV1. For the first SIB plan (Boost1), 33 and 25 Gy were specified to D95 of PTV1 and PTV2, respectively. For the second SIB plan (Boost2), 40, 33, and 25 Gy were specified to D95 of boostCTV, PTV1 and PTV2, respectively, and boostCTV was more prioritized than D95 of PTV1. For the third SIB plan (Boost3), 33 and 25 Gy were specified to D95 of PTV1 and PTV3, respectively. The dose constraints for OARs were as follows: V12 (volume receiving ≥ 12 Gy) of combined kidney less than or equal to 75%; the D50 of liver less than or equal to 12 Gy; the V23 to spinal cord less than 0.35 mL; the D9mL, D3mL, and D1mL of the GI‐OARs (stomach, duodenum, or bowel) less than or equal to 15 Gy, less than or equal to 20 Gy, and less than or equal to 33 Gy, respectively. We referred to the previous studies for these OAR constraints.^21^, ^22^ The calculation algorithm was Collapsed Cone v 2.3, and the calculation grid sizes were 0.2, 0.2, and 0.25 cm in left–right, anterior–posterior, and superior–inferior directions, respectively.

The following factors were evaluated. The target coverage and the dose to OARs in four SBRT plans (InitPlan, Boost1, Boost2, and Boost3), and the number of cases with one or more violation were evaluated. The statistical significance in the difference in OAR doses between InitPlan vs SIB‐SBRT plans were tested by a paired *t*‐test.

### 2.B Geometry‐driven DVH prediction model

#### 2.B.1 Framework of the predicting DVHs

**Figure 3:**
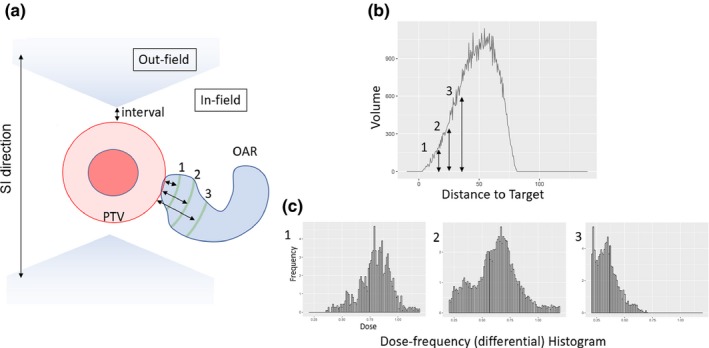
Framework of geometry‐driven dose–volume histogram prediction model. (a), (b) For each voxel of OARs, the minimum distance from PTV was calculated. Based on this distance, the voxels are sorted into different groups. The dose to voxels are acquired and combined to create dose–frequency histograms, such as (1), (2), and (3). In the models with “separate OARs,” the voxels within the infield and outfield spaces are regarded as different groups. (c) Each dose–frequency histogram contains the voxels and their doses at the same distant bins. These dose–frequency histograms were used to predict the dose‐to‐voxels at a certain distance for a new patient, by applying a median dose or a distribution of them.

The minimal Euclidean distance from each OAR voxel to the boundary of a target structure was calculated, and the voxels were sorted into bins based on their distance to a target volume (Fig. 3).^25^, ^26^, ^27^, ^28^ Here, we regarded the outermost target structure (i.e., PTV1 for InitPlan, PTV2 for Boost1 or Boost2, and PTV3 for Boost3) as the target volume. Then, we obtained a histogram of doses received by voxels (dose–frequency histogram) at the same distance bin. The dose to each voxel was normalized by dividing by the prescription dose to the outermost target. Based on a preliminary result, the width of a distance bin was set to 0.5 mm with a range between −10 mm and 150 mm. Then, all dose–frequency histograms at the same distance bin from the training cohorts were combined.

To approximate the dose distribution of each distance bin and apply doses to each voxel of an OAR in a new patient, two approaches were tested. First, the single median value of a dose–frequency histogram was used to represent a dose at a distance bin, and was applied to each voxel of an OAR of a new patient, as tested in a previous study for liver SBRT.^26^ Second, the distribution of dose–frequency histogram was directly applied to represent the distribution of those in voxels at the corresponding distance bin of a new OAR, to keep the characteristics of dose–frequency histograms from training cohorts. In addition, we tested the performance of dividing OAR voxels into separate volumes and obtaining two dose histograms: the dose histograms of voxels that are inside the treatment fields (infield) and those that are outside the treatment fields (outfield), as described previously.^25^, ^27^ The current study included both coplanar and noncoplanar beams (within ±30° of the axial plane), so that outfield regions became a double conical region in three‐dimensional space (Fig. 3). We tested three margin sizes (0, 5, and 10 mm) for the interval between the apex of each cone and the most superior/inferior aspects of the target surface.

#### 2.B.2 Evaluation and model validation experiments

First, we created the models using data from all 20 patients and predicted their DVHs. The performance of each model was evaluated by the mean and the standard deviation (SD) of the sum of residuals (SR) in each model.

$$SR=\sum_{D=0}^{\infty }\left(DVH(D)-DVH(D)pred\right)\cdot \Delta D$$

To capture the essential behavior of dose histograms in the training cohort, the absolute mean SR should be much less than that of the SD of SR.^25^ Next, the performance of models for the validation cohort was evaluated multiple times (25 in this study) in a twofold cross‐validation test (i.e., double cross‐validation test); we separated the cohorts into two groups that randomly included 10 patients each, and we used one group as a training cohort to create a prediction model and the other group as a validation cohort. Then, the prediction errors were assessed with the mean and SDs of SR and the sum of squared residuals (RSS).

$$RSS=\sum_{D=0}^{\infty }{\left((DVH(D)-DVH(D)pred)\cdot \Delta D\right)}^{2}$$

Because three DVH metrics (D1mL, D3mL, and D9mL) of GI‐OARs are important factors for the plan validity, we evaluated the accuracy (AC) in predicting these metrics with the following factors: the absolute difference in volume, the positive predictive value (PPV), the negative predictive values (NPV), and the AC for predicting the violation. AC is defined as the ratio of true positives and true negatives to total predictions. If the model properly predicts that the critical DVHs are violated in validation cases, PPV should become high; and if the model properly predicts that the critical DVHs are not violated, NPV should become high.

### 2.C Model‐based experiments

#### 2.C.1 Required distance to keep the prediction performance of the model

We expected that the dose histograms from the proximal part of an OAR are more related to the prediction of DVHs. To estimate the impact of the distance‐to‐target on the performance of DVH prediction, we limited the distance‐to‐target and simulated changing it from 5 to 50 mm, within which we could only use the dose–frequency histograms to predict DVHs. The model performance was assessed by the RSS.

#### 2.C.2 Simulations to assess the impact of proximity volumes of OARs on plan validity

By using our model, we assessed the impact of proximity volumes of GI‐OARs on the ratio of valid plans, which met all dose constraints. We simulated changing volumes of virtual GI‐OARs from 5 to 50 mL, which were located randomly according to a normal (Gauss) distribution with a mean of a certain distance‐to‐target. Because the presented model does not include GTV parameters, simulations on GTV parameters are not performed. Then, we predicted the DVHs with the prediction model and assessed the ratio of valid SBRT plans. The distance‐to‐target was selected as 0–5 mm to 0–60 mm with an interval of 5 mm, and the number of simulations was 250.

## 3 RESULTS

### 3.A DVH analysis for four SBRT plans

The mean PTV values are 59, 199, and 379 for PTV1, PTV2, and PTV3, respectively. Among 20 patients, 2 patients (cases #4 and #15) did not have a duodenum volume because of a prior surgical intervention. The results of DVH analyses are shown in Table 3. The target coverage was satisfied in all SBRT and SIB‐SBRT plans, but the doses to PTV2 or PTV3 slightly deviated from 25 Gy in three SIB‐SBRT plans because the prescription dose of 33 Gy to D95 of PTV1 (or 40 Gy to boostCTV in the plan Boost2) was prioritized, and we allowed the relaxed dose coverages for PTV2 or PTV3. Although PTV3 was not optimized to receive 25 Gy, the median dose (D50) to PTV3 in Boost1 or Boost2 was 23 or 25 Gy, respectively, which may partly satisfy the purpose of covering the elective nodal areas. The dose to CTVs were often modest when compared with dose coverages to the corresponding PTVs because CTVs had overlaps with GI‐OARs while the overlaps between PTVs and GI‐OARs were subtracted from PTVs. An example dose distribution of four SBRT plans are shown in Supporting Information Fig. S1. Also, example DVHs from InitPlan and Boost3 are shown in Supporting Information Fig. S2.

**Table 3:** Summary of DVHs in SBRT and SIB‐SBRT plans

Structure		Constraint	Plans InitPlan	Boost1	†	Boost2	†	Boost3	†
GTV	D95% [%]		100.4 (1.6) 100.7 [94, 103]	98.2 (7.7) 101.5 [86, 103]	0.042	105.3 (16.8) 109 [84, 121]	n.s.	99.2 (4.3) 100.4 [89, 104]	0.031
CTVgeom	D95% [Gy]		18.2 (2.1) 18 [15, 22]	22.1 (5.3) 23.7 [13, 28]	0.003	22.2 (6) 23.3 [12, 35]	0.006	22.8 (4) 21.2 [18, 31]	<0.001
CTVprop	D95% [Gy]		3.7 (4.6) 1.6 [0, 18]	6.9 (7.3) 3 [0, 25]	0.001	7.6 (8) 3 [0, 25]	<0.001	22.8 (5.2) 23.9 [11, 33]	<0.001
boostCTV	D95% [Gy]		28 (7.9) 32.2 [4, 34]	30.7 (4.6) 32.1 [13, 34]		40 (0) 40 [40, 40]		31.8 (1.9) 32.6 [28, 34]	
PTV1	D95% [%]	=100% *	100.0 (0) 100 [100, 100]	100 (0) 100 [100, 100]		107 (5.6) 106.2 [100, 121]		100 (0) 100 [100, 100]	
PTV2	D95% [Gy]	=25 Gy a	13.6 (1.9) 13.7 [10, 17]	26 (0.6) 26 [25, 27]	<0.001	26.5 (1.3) 26.4 [25, 31]	<0.001	24.2 (3) 24.5 [19, 31]	<0.001
	D50% [Gy]		27 (1.8) 26.7 [23, 30]	30 (1.1) 29.8 [29, 32]	<0.001	33.4 (1.6) 33.4 [31, 38]	<0.001	30.2 (1.8) 29.8 [28, 34]	<0.001
PTV3	D95% [Gy]	=25 Gy a	2.8 (3.6) 1.2 [0, 14]	5.6 (6.2) 2.2 [0, 21]	0.001	6.2 (6.9) 2.3 [0, 24]	<0.001	25.8 (1.8) 25.5 [24, 33]	<0.001
	D50% [Gy]		16.1 (8.2) 16.2 [1, 33]	23 (7.1) 25.3 [5, 33]	<0.001	25.4 (7.7) 26.7 [8, 40]	<0.001	29.1 (2.2) 28.5 [26, 35]	<0.001
Stomach	D1mL [Gy]	<33 Gy	18.2 (9.7) 20 [0, 34]	18.7 (7.2) 20.6 [0, 31]	n.s.	19.7 (7.5) 21.6 [0, 32]	n.s.	21.3 (6.5) 22.9 [1, 33]	0.035
	D3mL [Gy]	<20 Gy	15.3 (8.4) 15.2 [0, 31]	16.2 (6.2) 17.5 [0, 26]	n.s.	17.4 (6.7) 19.3 [0, 27]	n.s.	19.2 (6) 20.7 [1, 30]	0.003
	D9mL [Gy]	<15 Gy	11.4 (6.1) 11.5 [0, 23]	13.1 (5.2) 14.4 [0, 19]	0.032	14.2 (5.6) 15.7 [0, 20]	0.004	16.4 (5.4) 17.7 [1, 23]	<0.001
	Dmean [Gy]		2.2 (1.7) 2[0, 6]	3 (1.9) 2.8 [0, 7]	<0.001	3.4 (2.5) 3 [0, 10]	0.001	4.4 (2.2) 4.5 [0, 8]	<0.001
Duodenum	D1mL [Gy]	<33 Gy	19.8 (11.2) 19 [0, 35]	19.5 (9.4) 20.2 [0, 34]	n.s.	20.2 (9.1) 21.8 [0, 34]	n.s.	21.8 (9.1) 24 [0, 34]	n.s.
	D3mL [Gy]	<20 Gy	16.9 (10.4) 15.4 [0, 33]	16.7 (8.3) 17.2 [0, 31]	n.s.	17.5 (8.1) 18 [0, 32]	n.s.	19.7 (8.5) 20.2 [0, 32]	0.07
	D9mL [Gy]	<15 Gy	11.7 (8.8) 11.6 [0, 32]	12 (6.8) 13.5 [0, 26]	n.s.	12.9 (7.3) 13.9 [0, 26]	n.s.	16.6 (7.2) 16.7 [0, 28]	0.004
	Dmean [Gy]		6.5(5.5) 5.4[0, 19]	7.2 (5.4) 6.7 [0, 22]	n.s.	8 (6.1) 7.6 [0, 24]	0.047	11.3(5.7)12.2[0,23]	<0.001
Small bowel	D1mL [Gy]	<33 Gy	17 (6) 17.6 [6, 28]	20.3 (5.4) 20.1 [11, 30]	0.001	21.1 (4.8) 21.6 [11, 30]	<0.001	23.6 (3.8) 24.4 [14, 29]	<0.001
	D3mL [Gy]	<20 Gy	13.8 (5) 13.9 [3, 23]	17.2 (4.6) 17.9 [8, 25]	<0.001	18.1 (4.4) 19.4 [8, 25]	<0.001	20.9 (3.9) 21.9 [10, 26]	<0.001
	D9mL [Gy]	<15 Gy	10.5 (4.3) 11.4 [2, 18]	13.1 (4.3) 14.3 [5, 21]	<0.001	14.1 (4.7) 15.3 [5, 22]	<0.001	17.6 (3.9) 17.9 [6, 23]	<0.001
	Dmean [Gy]		2 (1.7) 1 [0, 7]	2.6 (2.1) 1.7 [0, 9]	<0.001	2.9 (2.1) 2 [0, 9]	<0.001	4.5 (2.4) 4 [1, 9]	<0.001
Lt. Kidney	V12Gy [%]	<75% b	0.6 (1.5) 0[0, 6]	4.1 (7.7) 0 [0, 30]	0.032	4.3(6.7)0.4[0,22]	0.008	11.4(14.1)5.2[0,52]	0.002
Rt. Kidney	V12Gy [%]	<75% b	3.5 (8.8) 0[0, 33]	8.6(15.9) 1.6[0, 62]	0.039	10.3(18.4)1[0,67]	0.03	13.5(10.1)13.7[0,41	<0.001
Liver	D50% [Gy]	<12 Gy	0.6 (0.5) 0[0, 2]	1.1 (0.9) 0.9[0, 3]	<0.001	1.2 (1) 1 [0, 3]	<0.001	2.1 (1.3) 1.8 [0, 6]	<0.001
Spinal Cord	V8Gy [mL]		1.3 (2.1)0 [0, 8]	2 (2.4) 0.8 [0, 7]	n.s.	3.1 (3.4) 2 [0, 11]	0.003	6.1 (4.1) 7.2 [0, 15]	<0.001
	V23Gy[mL]	<0.35 mL	0 (0) 0 [0, 0]	0 (0) 0 [0, 0]	n.s.	0 (0) 0 [0, 0]	n.s.	0 (0) 0 [0, 0]	n.s.

Abbreviations: Dxx, the dose covering xx (% or mL) of the volume; Vxx, the volume receiving xx Gy; Dmean, the mean dose; Lt., left; Rt., right; GTV, gross tumor volume; CTV, clinical target volume; CTVgeom, geometrical CTV (i.e. GTV plus 10–15 mm margin); CTVprop, prophylactic CTV (i.e. GTV plus a prophylactic nodal areas); PTV, planning target volume; n.s., not significant.

All data are shown in the mean, (standard deviation), median, and [min, max] from 20 patients. PTV1, PTV2, and PTV3 are defined as a 3‐mm isotropic expansion of the GTV, CTVgeom, and CTVprop, respectively, and the overlaps between PTVs and OARs are subtracted.

a = PTV coverage was compromised if OAR constraints could not be met. b = combined kidney

* 40 Gy was prescribed to D95 of boostCTV in plan Boost2, while 33 Gy was prescribed to D95 of PTV1 in the other plans. Also, 25 Gy was prescribed to D95 of PTV2 in Boost1 and Boost2 or PTV3 in Boost3, respectively.

† *P* values were calculated by paired t‐test comparing InitPlan with Boost1, Boost2, or Boost3.

Overall dose increases were observed in GI‐OARs in Boost1, Boost2, and Boost3 when compared with InitPlan (Table 3). The statistical significances were distinct among three SIB‐SBRT plans. The increase in high‐dose volumes (D1–3mL) of the stomach and duodenum was seen in Boost1 or Boost2, but was not statistically significant (displayed as “n.s.” in Table 3). Although a significant increase in low‐dose volume (D9mL) of the stomach (*P* = 0.032 and 0.004) and duodenum and in all DVH metrics of the small bowel were seen in both of these two SIB‐SBRT plans (all *P* values equal or less than 0.001), the mean DVH metrics did not exceed the dose constraints. In contrast, all DVH metrics of GI‐OARs in Boost3 significantly increased from InitPlan, and most of the mean values exceeded the dose constraints (Table 3).

Among the four SBRT plans, violations were observed in 6 cases, 8 cases, 8 cases, and all 20 cases in InitPlan, Boost1, Boost2, and Boost3, respectively. All violations were observed regarding the constraints for the stomach, duodenum, or small bowels, whereas no violation was observed regarding the other OARs.

### 3.B Performance of DVH prediction

First, all patient data were used to develop prediction models and the predicted DVHs were analyzed. Table 4 and Supporting Information Fig. S3 show the SRs regarding DVHs for the stomach, duodenum, and small bowel, demonstrating that all models achieved mean SR much smaller than their SDs. This implies that all models capture the essential behavior of the training set. Compared with the models in which the median value represents the doses of a distance bin (“Median” in Table 4), the models in which the distribution of dose–frequency histograms were directly used for the dose–frequency histogram at a corresponding distance bin (“Distribution” in Table 4) showed the lower mean and SDs of SRs; thus, the latter models are deemed to be the better ones.

**Table 4:** Sum of residuals between the planned and predicted dose–volume histograms in the training cohorts

PLAN	OAR^b^	Median^a^			Distribution^a^		
		Stomach	Duodenum	Bowel	Stomach	Duodenum	Bowel
InitPlan	A	0.018 (0.023)	0.033 (0.049)	0.027 (0.036)	0.001 (0.022)	0.009 (0.048)	0.005 (0.033)
	B	0.018 (0.022)	0.012 (0.044)	0.014 (0.027)	0.004 (0.019)	0.006 (0.044)	−0.001 (0.025)
	C	0.009 (0.022)	0.007 (0.045)	0.008 (0.027)	−0.006 (0.02)	−0.002 (0.045)	−0.006 (0.026)
	D	0.014 (0.022)	0.015 (0.048)	0.025 (0.034)	−0.001 (0.02)	0.004 (0.045)	0.01 (0.031)
Boost1	A	0.009 (0.048)	0.041 (0.095)	0.032 (0.063)	−0.013 (0.047)	0.016 (0.094)	0.005 (0.064)
	B	0.016 (0.045)	0.026 (0.098)	0.022 (0.054)	−0.006 (0.042)	0.002 (0.101)	0.006 (0.052)
	C	0.035 (0.046)	0.001 (0.097)	0.027 (0.057)	0.004 (0.043)	−0.013 (0.098)	0.005 (0.055)
	D	−0.011 (0.047)	0.035 (0.095)	0.022 (0.058)	−0.027 (0.044)	0.014 (0.097)	0.007 (0.056)
Boost2	A	0.014 (0.035)	0.061 (0.07)	0.032 (0.05)	−0.007 (0.033)	0.026 (0.072)	0.004 (0.05)
	B	0.018 (0.032)	0.042 (0.09)	0.01 (0.041)	0.002 (0.029)	0.028 (0.079)	−0.007 (0.042)
	C	0.02 (0.032)	0.018 (0.077)	0.016 (0.045)	0.001 (0.029)	0.015 (0.075)	−0.004 (0.045)
	D	0.013 (0.033)	0.051 (0.083)	0.03 (0.049)	−0.002 (0.03)	0.031 (0.076)	0.011 (0.048)
Boost3	A	0.024 (0.052)	0.051 (0.096)	0.037 (0.054)	0.001 (0.051)	0.018 (0.099)	0.004 (0.054)
	B	0.024 (0.048)	0.04 (0.112)	0.012 (0.045)	0.004 (0.045)	0.028 (0.102)	−0.012 (0.046)
	C	0.034 (0.05)	0.022 (0.103)	0.021 (0.048)	0.01 (0.047)	0.014 (0.102)	−0.005 (0.048)
	D	0.026 (0.049)	0.059 (0.109)	0.035 (0.052)	0.007 (0.047)	0.041 (0.103)	0.012 (0.051)

Sum of residuals (SR) are shown as mean (and standard deviation) from all 20 patients. All 20 cases are used as a single training cohort for the prediction models, and the prediction errors for actual DVHs from all 20 cases are tested.

a The dose to voxels is represented by the “Median dose” or “Distribution” from dose–frequency histograms at a corresponding distance bin.

b A = single OAR, B = separated OAR with a 0‐mm interval, C = separated OAR with a 5‐mm interval, and D = separated OAR with a 10‐mm interval.

Next, half of the patient data were used for developing the model to predict DVHs in the other patients, and the double cross‐validation test was performed. Table 5 and Supporting Information Fig. S4 show the resulting SRs of models in different settings. The mean and SDs of SRs are almost equivalent among the models, whereas the models with separate OAR volumes (Fig 3, infield and outfield) and the shorter interval between PTV and the apex of the double cone had better performance than those with longer intervals according to RSS; the overall averaged RSSs from three GI‐OARs were 9.9 × 10e‐6 for single OAR models, and 7.9 × 10e‐6, 8.3 × 10e‐6, and 9.0 × 10e‐6 for separate OAR volume models with 0‐, 5‐, and 10‐mm intervals, respectively. Thus, the separate OAR volume model with the 0‐mm interval is deemed to be the best performance.

**Table 5:** Sum of residuals between the planned and predicted dose–volume histograms

PLAN	OAR^a^	Stomach	Duodenum	Bowel	Lt. kidney	Rt. kidney	Liver	Cord
InitPlan	A	0.001 (0.024)	0.006 (0.052)	0.001 (0.034)	−0.004 (0.045)	−0.002 (0.05)	0.000 (0.025)	0.000 (0.024)
	B	0.001 (0.021)	0.009 (0.048)	−0.001 (0.027)	−0.002 (0.038)	−0.004 (0.05)	0.000 (0.02)	0.000 (0.023)
	C	0.001 (0.021)	0.009 (0.048)	−0.001 (0.028)	−0.003 (0.038)	−0.001 (0.051)	0.000 (0.021)	0.000 (0.023)
	D	0.001 (0.022)	0.009 (0.049)	0.000 (0.032)	−0.003 (0.04)	−0.002 (0.049)	0.000 (0.021)	0.000 (0.023)
Boost1	A	−0.005 (0.051)	−0.001 (0.105)	0.003 (0.063)	−0.001 (0.073)	−0.006 (0.064)	0.000 (0.042)	−0.001 (0.032)
	B	−0.005 (0.045)	−0.002 (0.106)	0.002 (0.053)	−0.002 (0.058)	−0.005 (0.061)	0.001 (0.031)	−0.002 (0.03)
	C	−0.005 (0.047)	0.000 (0.105)	0.002 (0.055)	−0.003 (0.058)	−0.006 (0.065)	0.001 (0.035)	−0.001 (0.03)
	D	−0.005 (0.047)	−0.005 (0.108)	0.002 (0.059)	−0.004 (0.072)	−0.005 (0.064)	0.000 (0.034)	−0.001 (0.031)
Boost2	A	0.000 (0.034)	0.02 (0.081)	0.002 (0.051)	−0.003 (0.074)	0.002 (0.068)	0.002 (0.042)	−0.001 (0.028)
	B	0.000 (0.03)	0.023 (0.077)	−0.002 (0.045)	−0.001 (0.055)	0.002 (0.063)	0.004 (0.029)	−0.002 (0.026)
	C	0.000 (0.032)	0.023 (0.078)	−0.001 (0.047)	−0.004 (0.057)	0.004 (0.064)	0.003 (0.031)	−0.002 (0.027)
	D	0.001 (0.033)	0.025 (0.079)	−0.001 (0.051)	−0.004 (0.06)	0.002 (0.062)	0.003 (0.031)	−0.001 (0.027)
Boost3	A	0.007 (0.052)	0.019 (0.113)	0.000 (0.054)	−0.003 (0.072)	0.002 (0.064)	0.002 (0.045)	0.001 (0.044)
	B	0.007 (0.047)	0.024 (0.105)	−0.003 (0.049)	−0.001 (0.051)	0.002 (0.06)	0.004 (0.031)	0.001 (0.042)
	C	0.007 (0.048)	0.022 (0.107)	−0.003 (0.051)	−0.003 (0.052)	0.004 (0.06)	0.004 (0.032)	0.000 (0.044)
	D	0.008 (0.05)	0.024 (0.106)	−0.002 (0.055)	−0.003 (0.056)	0.003 (0.059)	0.004 (0.032)	0.001 (0.042)

Sum of residuals (SR) are shown as mean (and standard deviation) from all 20 patients. Half of 20 cases are used as a single training cohort for the prediction models, and the prediction errors for actual DVHs from the other cases are tested. This validation is repeated for 25 times and SRs are averaged out.

a A = single OAR, B = separated OAR with a 0‐mm interval, C = separated OAR with a 5‐mm interval, and D = separated OAR with a 10‐mm interval.

The median absolute errors for stomach were 0.1, 1.0, and 3.3 mL for V33, V20, and V15, respectively. Those for duodenum were 0.0, 1.0, and 2.0 mL, respectively. Those for small bowel were 0.0, 1.0, and 3.1 mL, respectively. Supporting Information Fig. S5 represents sample comparisons from two cases between the predicted DVHs and the actual DVHs from the validation set. The positive predictive value (PPV) in predicting one or more violations in critical DVH metrics (D9mL, D3mL, and D1mL) in GI‐OARs was 66% (3055/4650), and the negative predictive value (NPV) was 90% (11 505/12 750). The overall AC in predicting the valid or invalid plans was 84% (14 560/17 400), demonstrating a feasibility in identifying the plan validity. The SRs for the other OARs (kidneys, liver, and spinal cord) are also shown in Table 5 and Supporting Information Fig. S4. All models achieved mean SRs much smaller than their SDs. Because no violations were observed in these OARs (Table 3), evaluating predictive performance of DVH metrics was avoided.

### 3.C Required distance‐to‐target for the prediction model

**Figure 4:**
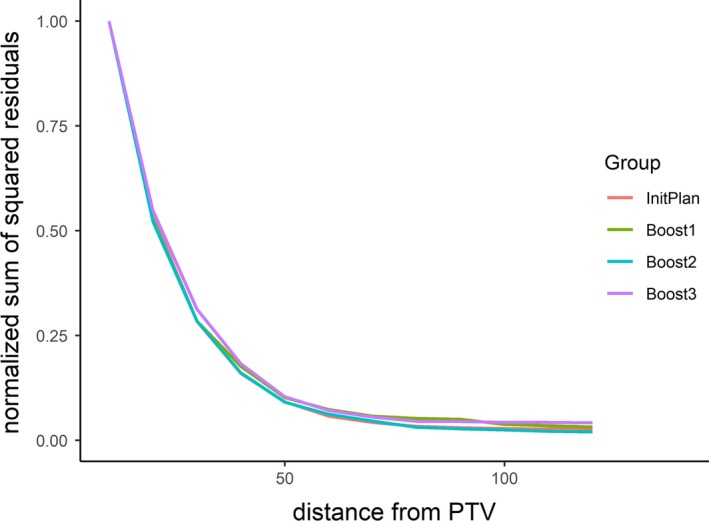
Impact of distance‐to‐target on the performance of the prediction model. The averaged sum of squared residuals (RSS) between the actual and predicted dose–volume histograms in all three gastrointestinal organs at risks from 20 patients are shown. The RSSs are normalized by dividing with the RSS at distance‐to‐target = 5 mm (minimum).

Figure 4 illustrates the RSSs as a function of distance‐to‐target, only within which we could use the dose histograms to develop the prediction model. As the distance‐to‐target increases, the RSS decreases in all models. The reduction of RSS was slowed over the distance of 40–50 mm, suggesting that the performance of the prediction models within the distance of 40–50 mm would be comparable to those with the whole (150 mm) distances.

### 3.D Impact of proximity volumes of GI‐OARs on the plan validity

**Figure 5:**
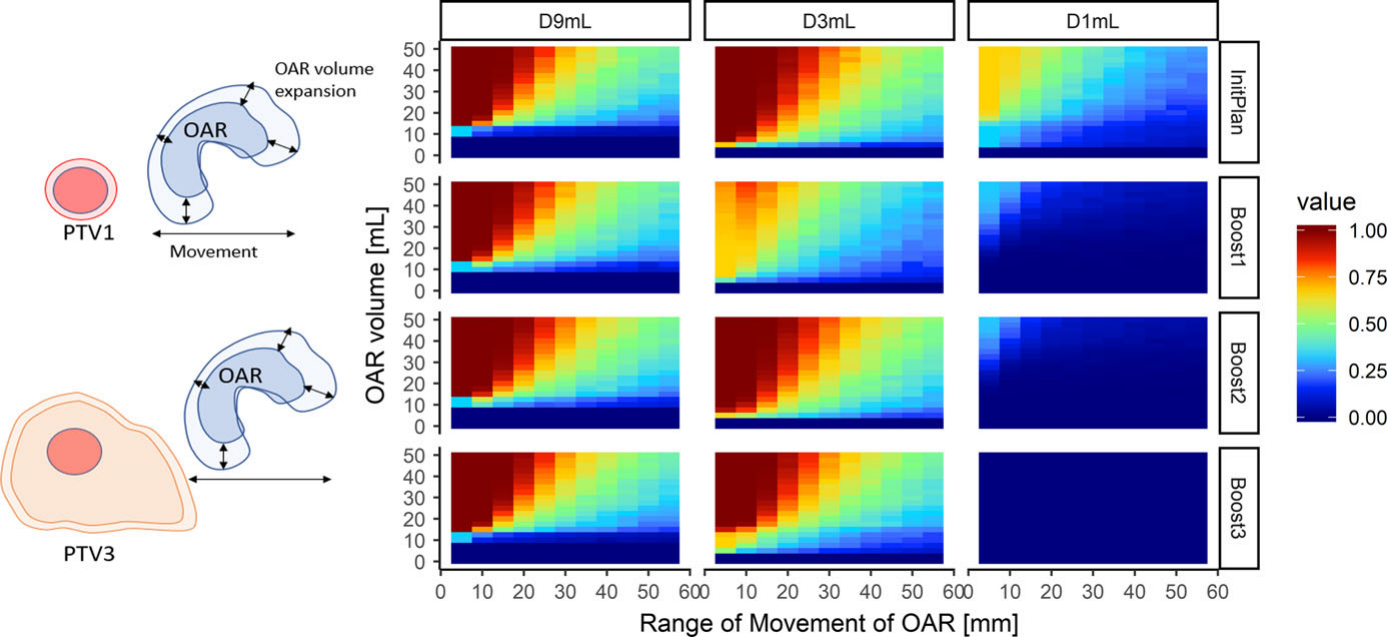
Impact of proximity volumes and distances of gastrointestinal (GI)‐organs at risk (OAR) on plan feasibility. The probability of planning failure is estimated by simulating a virtual GI‐OAR to move and change its position and size. The Y axis shows the size of gastrointestinal (GI)‐organs at risk (OAR), and the X axis shows the range of position of GI‐OAR. The red color represents that the planning failure ratio is high, and blue is low. Each column represents the results of 250 situations of randomly located GI‐OAR.

Supporting Information Fig S6 illustrates the median (25th–75th percentiles) of GI‐OAR volumes from the 20 patients in the current study according to the distances from the target. We sought to evaluate the impact of the proximity volumes of GI‐OARs on the ratio of plan validity by simulations in which virtual GI‐OARs were moved and changed their positions and sizes. Figure 5 shows the ratio of violations of DVH metrics. Compared with the D9mL and D3mL, the violations of D1mL were not widely observed. These differences in the four plans can be intuitively appreciated because three SIB plans had larger target volume and the prescription dose to D95 of PTV2 or PTV3 was 25 Gy, which was far below 33 Gy. It is estimated that the prescription dose of 33 Gy for PTV1 or 40 Gy for boostCTV did not substantially interfere in avoiding violations of V33 even with the proximity GI‐OARs. In contrast, when proximity GI‐OAR of approximately 10 mL or more volumes were located within 10–20 mm from the target volume, the violations for D3mL or D9mL were observed in most cases.

## 4 DISCUSSION AND CONCLUSION

Intensive efforts have been devoted for including SBRT in neoadjuvant settings of pancreatic cancer, but the prophylactic nodal irradiation has usually been avoided, and direct comparative studies of SBRT and SIB‐SBRT with prophylactic nodal areas have not yet been explored. This study is, to the best of our knowledge, the first study to focus on the detailed analysis of dosimetric feasibility of SBRT with prophylactic irradiation when administering SBRT for pancreatic cancer, in accordance with the recent dose/fractionation regimens as well as normal tissue constraints that are widely adopted in the modern clinical studies. We conducted this study for establishing the SBRT with prophylactic irradiation concept before its introduction to the clinical setting.

We performed retrospective treatment planning and comparisons of four SBRT plans to assess the planning feasibility in each SBRT scenario, and relevant questions are answered. SIB‐SBRT plans allow for inclusion of the prophylactic target volumes at the expense of dose increase to OARs. Overall, conventional SBRT (InitPlan) and experimental SIB‐SBRT with geometrical CTV (Boost1 and Boost2) satisfied all dose constraints in 63% of cases in the current study, whereas the results of including all prophylactic lymphatics (Boost3) were discouraging because no patient satisfied all dose constraints for GI‐OARs. This indicates that including a part of the prophylactic area is dosimetrically applicable, whereas the inclusion of the whole prophylactic nodal area cannot be a pragmatic approach. In addition, the inclusion of margin intensive boost yielded mostly equivalent results to Boost2, indicating that a boosting dose (40 Gy) to the margin of involved vasculature does not substantially increase GI‐OAR doses if the boosting area is away from GI‐OARs (1 cm in the current study). This result agrees with recent investigations on this strategy, opening the way for achieving a greater regression in this critical area of the tumor.^4^, ^5^, ^29^

The disappointing results of Boost3 had been expected given that the significantly larger volumes of prophylactic area (CTVprop and PTV3) yielded large overlap or proximity volumes of GI‐OARs around the target structures (Supporting Information Fig S6). All violations were regarding dose constraints of GI‐OARs, which were confirmed to be crucial for the successful planning. Also, these violations of constraints of GI‐OARs can be attributed to intraoperative complications that we reported previously.^15^, ^16^ Based on a preliminary investigation, it was suggested that the dose coverage for the whole prophylactic nodal area needed to be relaxed at least from D95% to D90%, or 25 to 23 Gy or less. Also, the retropancreatic space as well as the para‐aortic region between the root of SMA and celiac trunk were supposed to be easier to cover due to an anatomical nature of GI‐OARs. However, these suggestions were not conclusive because the individual planning feasibility was highly affected by the prescription dose and the target regions (and the anatomical feature). In contrast, compared with our previous studies,^15^, ^16^ InitPlan, Boost1, and Boost2 approaches in the current study achieved the considerably lower mean doses to GI‐OARs; the mean doses to the stomach and small bowel were 3.0–3.4 Gy and 2.6–2.9 Gy in the current study vs 7.8 and 6.7 Gy in the previous study.^15^ This would result in an acceptable toxicity even when SIB‐SBRT concepts are used in the clinical scenario.

To elucidate how patient anatomy influenced the plan feasibility, the geometry–dosimetry relationship was focused on in the current study. Our DVH prediction method uses the geometry–dosimetry information in previous patients to predict DVHs based on the individual anatomy in new patients, and we demonstrated that our model has a high performance for predicting DVHs. Among previous studies of geometry‐driven DVH estimation,^25^, ^26^, ^27^, ^28^, ^30^ two studies investigated pancreatic SBRT: Petit et al.^27^ used overlap volume histograms in prior patients who had an OAR that was more difficult to spare, and approximated the minimal achievable doses to liver and kidney in a new patient and identified possible candidates for replanning. More recently, Campbell et al. studied the artificial neural network dose models (ANN‐DMs)^31^ for pancreatic SBRT using data from 43 patients, and demonstrated that the mean absolute dose errors were less than or close to 5% at all distances from PTV.^28^ Our results add to these evidences in that the geometry‐driven DVH prediction can be achievable to SBRT with prophylactic area irradiation using a relatively simple model suggested in the current study, and the model can secure its performance using the dose–geometry data only within 40–50 mm from the target. The unique contribution of our model is to demonstrate the predictive performance of violations of specific DVH metrics in GI‐OARs in SBRT/SIB‐SBRT plans. We also demonstrated the difficulty in achieving a valid plan when GI‐OARs had larger volume or closer distance from the target volume through simulations of random positioning of virtual GI‐OARs (Fig. 5). This observation supports and can serve as a generalization of an idea of the overlapping volume between GI‐OARs and the expanded PTV and its cutoff value, which determines feasibility in achieving dose constraints as previously described by Yang et al.^4^ The results imply that the geometry defines a large portion of planning feasibility, and strongly support a conventional recognition that the planning feasibility relies on a favorable geometry between the GI‐OARs and the target volume.

Then, important questions remain as to how SBRT or SIB‐SBRT should be offered to the cases with large proximity GI‐OARs volume. The difficulty in achieving all dose constraints in pancreatic SBRT has been recognized,^4^, ^5^ and despite the differences in the planning techniques and dose constraints, we also showed a modest successful planning ratio of 65%. This could be because of the strict dose constraints in OARs and target coverage (D95 of PTV1) as well as the importance of individual patient anatomical factors as demonstrated in the current study. To offer SBRT and SIB‐SBRT strategy to a greater patient population, pragmatic approaches currently can be applicable: (A) using an increased treatment fractionation, (B) an allowance of compromised target coverage as used in the modern clinical studies,^21^ and (C) separating PTV volume into subvolumes to deliver a modest dose to overlap regions with OARs or PRVs.^32^, ^33^, ^34^, ^35^ Further investigation is required for the clinical influences of the compromises in the target coverage.

We admit that there are several limitations for interpreting our results. We did not utilize volumetric modulated arc (VMAT) techniques, which were reported favorably in several studies with conventional fractionation.^36^, ^37^, ^38^, ^39^ This is because, depending on institutional policy, we had not yet started to use VMAT for pancreas SBRT in clinical practice and in a preliminary analysis we found it difficult to meet the hard constraints. We observed that VMAT tended to spread out roundly the higher isodose lines around PTVs more than IMRT, which might have resulted in failure to achieve PTV1 coverage and GI‐OAR dose constraints simultaneously. For these cases, IMRT resulted in better results. Complex target structures in the current study as well as the strict dose constraints of GI‐OARs might be reasons for the difficulty, as a previous study also reported a similar trend.^3^ Also, while our model was comprised relatively simple parameters and little computational burden, one possible disadvantage of our model was that it does not predict a dose at each single voxel and therefore it cannot visualize the predicted dose distribution on three‐dimensional space. Because the focuses were to evaluate the feasibility of and to elucidate geometry–dosimetry relationship in SIB‐SBRT concepts for pancreatic cancer, the visualization of three‐dimensional point doses was abandoned in this study.

In summary, the planning feasibility of the SIB‐SBRT concept to include the a prophylactic area in pancreatic cancer was tested in this study. SBRT plans with geometrically defined CTV possibly can be treated with SIB‐SBRT techniques, but including all nodal areas with 25 Gy in five fractions seems unrealistic. The geometry‐driven DVH prediction model showed the strong geometry–dosimetry relationship in pancreatic SBRT, and alternative utilization of these models was demonstrated to assess the violations based on the patient geometry. Further studies are needed to define a role of SIB‐SBRT in the clinical setting for pancreatic cancer, and future prospective studies should aim to clarify the feasibility and tolerability of SIB‐SBRT with limited prophylactic regions.

## CONFLICTS OF INTEREST

The authors have no relevant conflicts of interest to disclose.

## Supporting information


**Fig. S1.** Representative dosimetry for SBRT and SIB‐SBRT plans in (a) InitPlan, (b) Boost3, (c) Boost1, and (d) Boost2. The 120% (yellow), 100% (orange), 95% (light blue), 90% (purple), 80% (blue), 70% (green), 60% (light green), and 50% (dark blue) isodose lines are depicted. The GTV is depicted as a red line. The CTVgeom is depicted as a yellow line in (c) and (d), and boostCTV is depicted as a purple line in (d). The CTVprop is depicted as an orange line in (b).

**Fig. S2.** Sample dose–volume histogram (DVHs) for SBRT plan and SIB‐SBRT plans in (a) InitPlan and (b) Boost3. The DVH curves are calculated by averaging the DVHs from 20 patients in these two SBRT strategies.

**Fig. S3.** Sum of residuals between the planned and predicted dose–volume histogram (DVHs) in the training cohorts. All 20 cases are used as a single training cohort and the prediction errors from the actual DVHs are shown as absolute values. The errors are significantly lower in the “Distribution” models compared with the “Median” models.

**Fig. S4.** Sum of residuals between the planned and predicted dose–volume histograms (DVHs). Half of 20 cases are used as a single training cohort for building a prediction model and the prediction errors from the actual DVHs from the other cases are assessed. The results of errors are fluctuated between the model settings.

**Fig. S5.** Sample dose–volume histograms (DVHs) for three GI‐OARs from two patients (cases #11 and 14). The prediction model was generated from 10 cases (#1–10), and the DVHs in these patients are predicted. The blue line represents the actual DVH and the yellow line represents the DVHs predicted with the geometry‐driven model.

**Fig. S6.** Overlapping volume between GI‐OARs and the expanded target volume are shown. As the size of expanded target volume (distance‐to‐target) increases, the overlapping volume increases.
