# Supplementary figures and images for: Elective nodal irradiation with simultaneous integrated boost stereotactic body radiotherapy for pancreatic cancer: Analyses of planning feasibility and geometrically driven DVH prediction model
Source: J Appl Clin Med Phys. 2019 Jan 13;20(2):71–83. doi: 10.1002/acm2.12528 (PMC6370996; doi:10.1002/acm2.12528)

Sup Fig S1

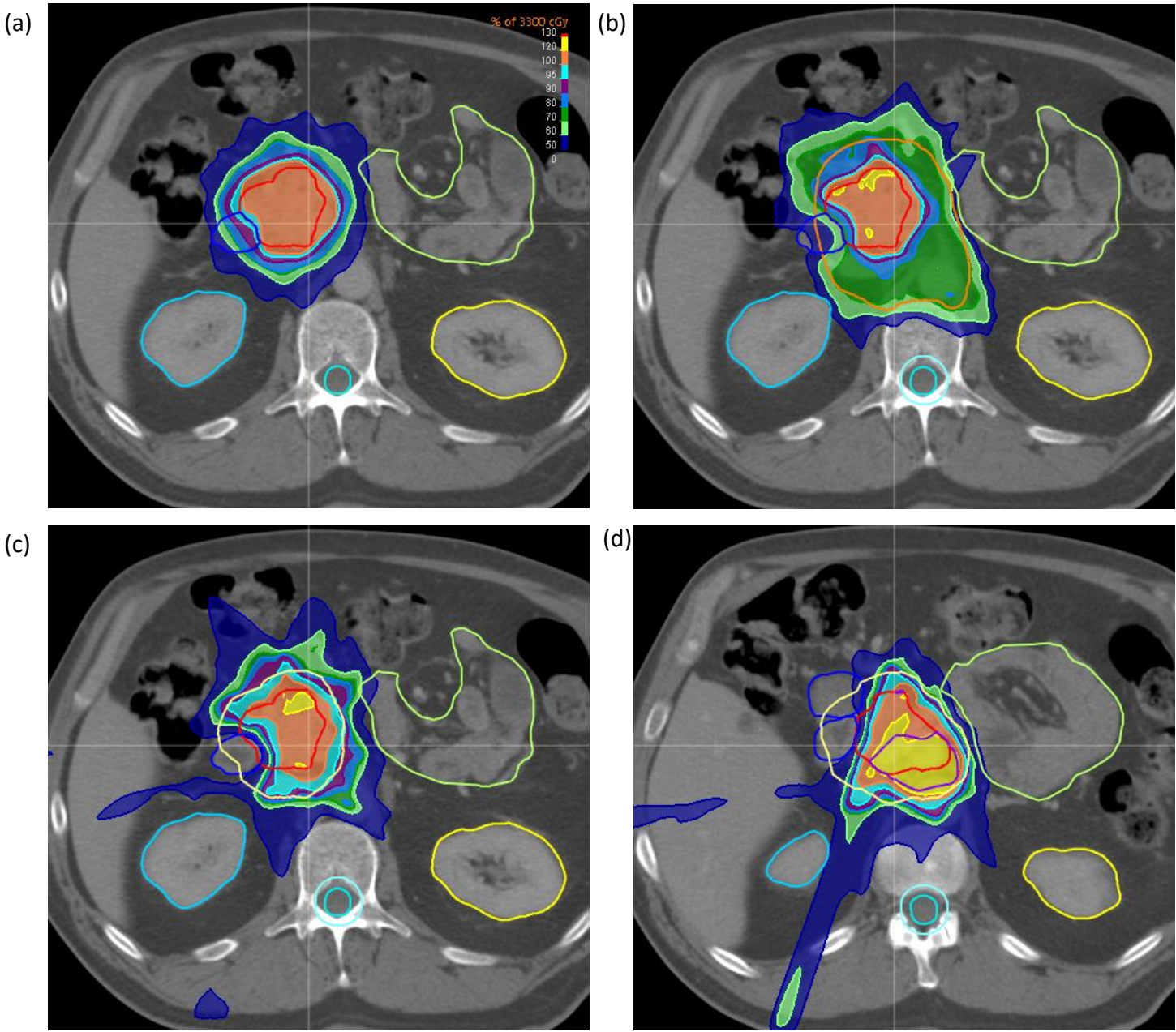

Supplement: Supplementary file 1 — Fig. S1. Representative dosimetry for SBRT and SIB‐SBRT plans in (a) InitPlan, (b) Boost3, (c) Boost1, and (d) Boost2. The 120% (yellow), 100% (orange), 95% (light blue), 90% (purple), 80% (blue), 70% (green), 60% (light green), and 50% (dark blue) isodose lines are depicted. The GTV is depicted as a red line. The CTVgeom is depicted as a yellow line in (c) and (d), and boostCTV is depicted as a purple line in (d). The CTVprop is depicted as an orange line in (b). [file ACM2-20-71-s001.pdf]

Sup Fig S2

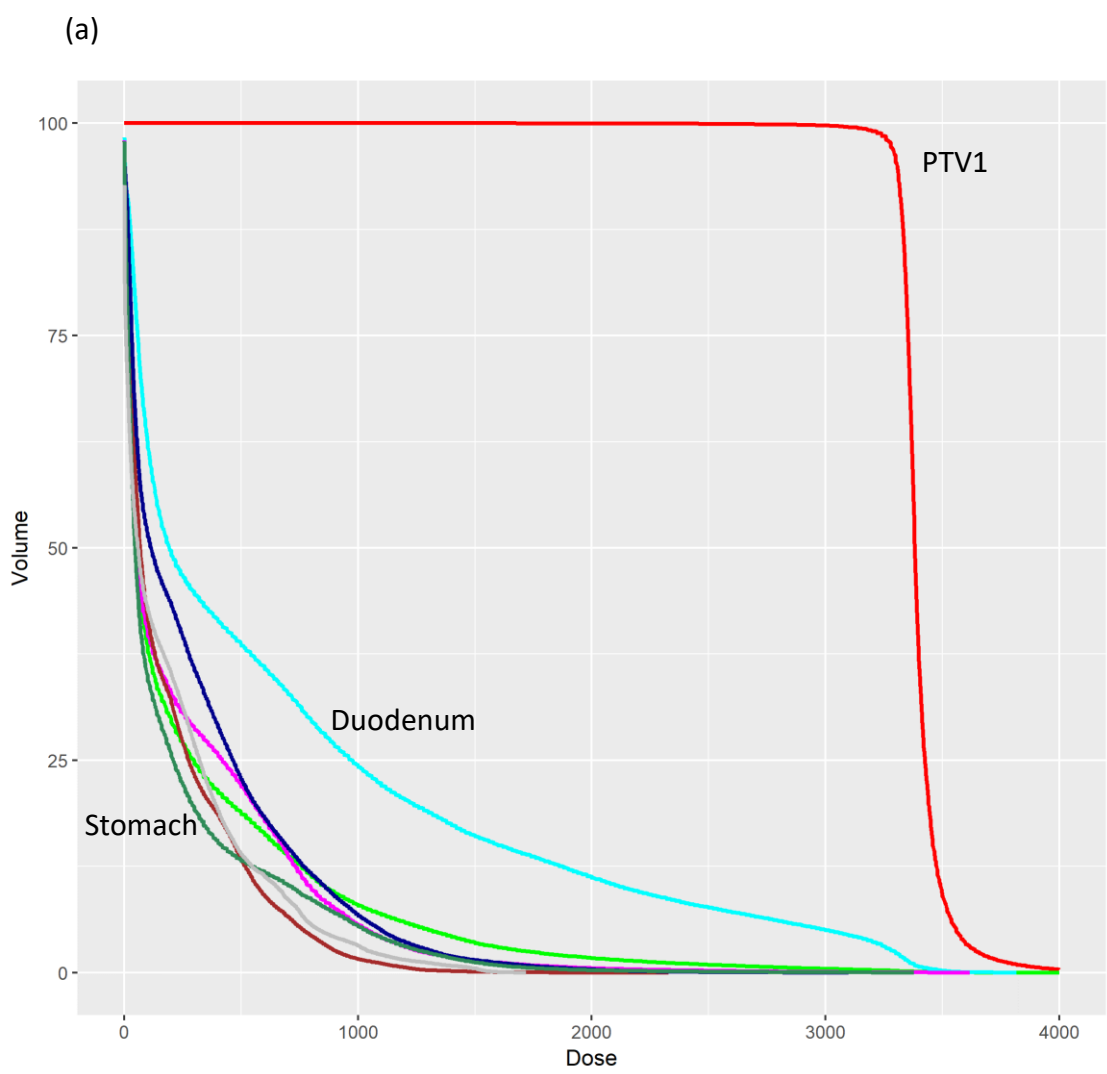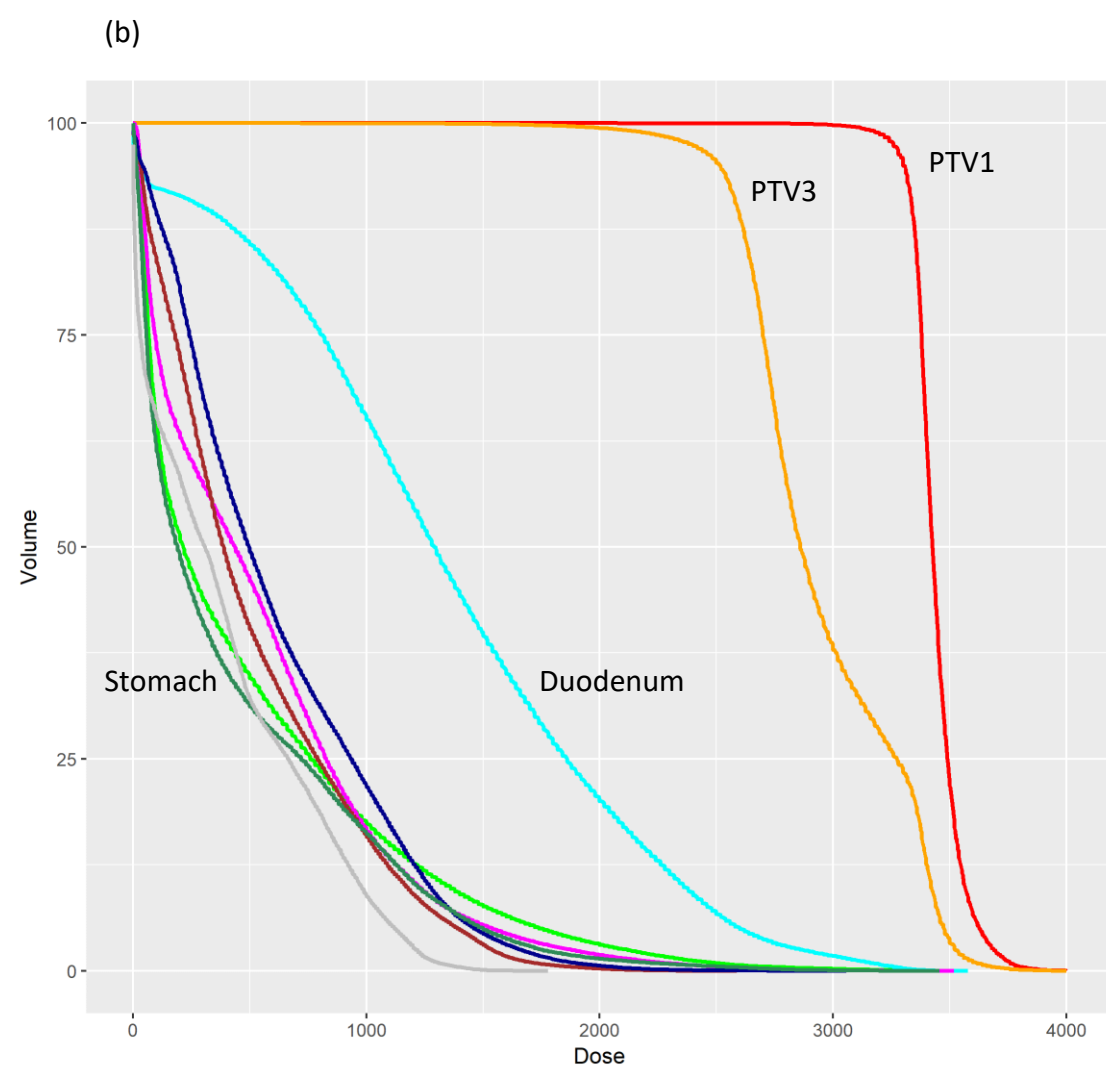

Supplement: Supplementary file 2 — Fig. S2. Sample dose–volume histogram (DVHs) for SBRT plan and SIB‐SBRT plans in (a) InitPlan and (b) Boost3. The DVH curves are calculated by averaging the DVHs from 20 patients in these two SBRT strategies. [file ACM2-20-71-s002.pdf]

Sup Fig S3

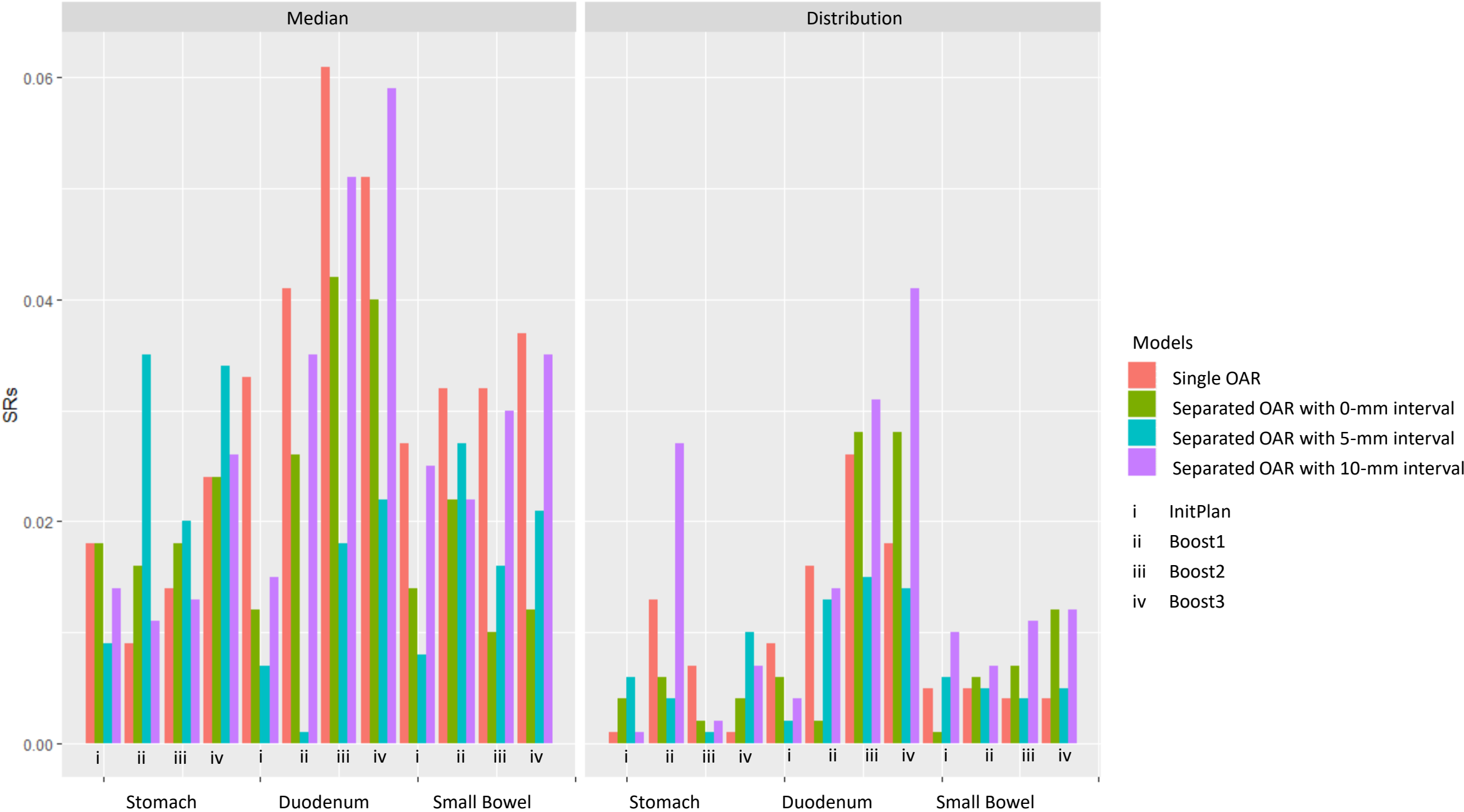

Supplement: Supplementary file 3 — Fig. S3. Sum of residuals between the planned and predicted dose–volume histogram (DVHs) in the training cohorts. All 20 cases are used as a single training cohort and the prediction errors from the actual DVHs are shown as absolute values. The errors are significantly lower in the “Distribution” models compared with the “Median” models. [file ACM2-20-71-s003.pdf]

Sup Fig S4

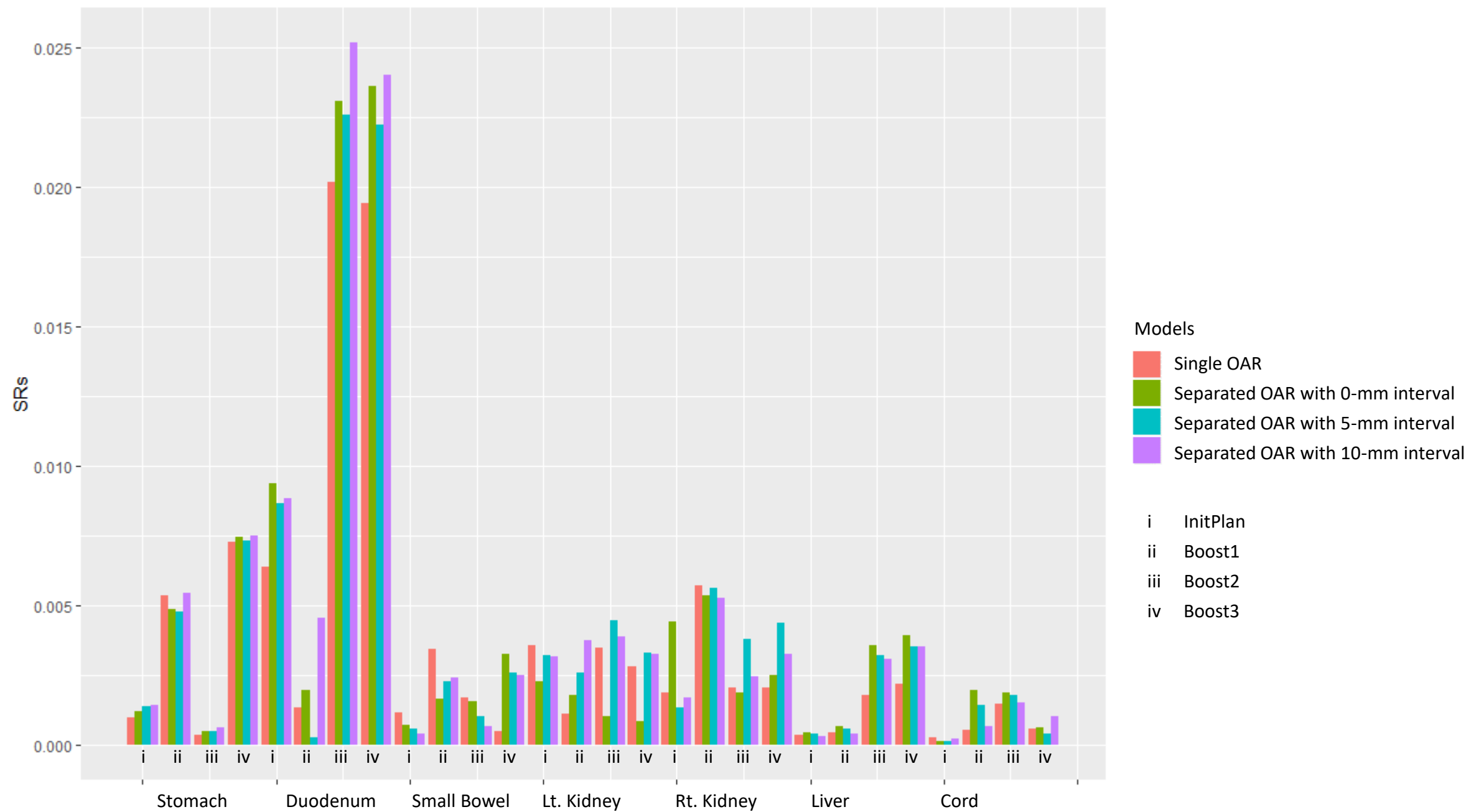

Supplement: Supplementary file 4 — Fig. S4. Sum of residuals between the planned and predicted dose–volume histograms (DVHs). Half of 20 cases are used as a single training cohort for building a prediction model and the prediction errors from the actual DVHs from the other cases are assessed. The results of errors are fluctuated between the model settings. [file ACM2-20-71-s004.pdf]

Sup Fig S5

Case#11

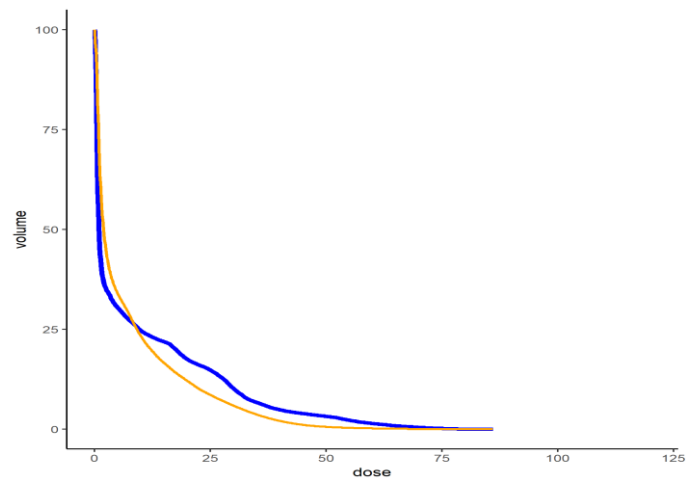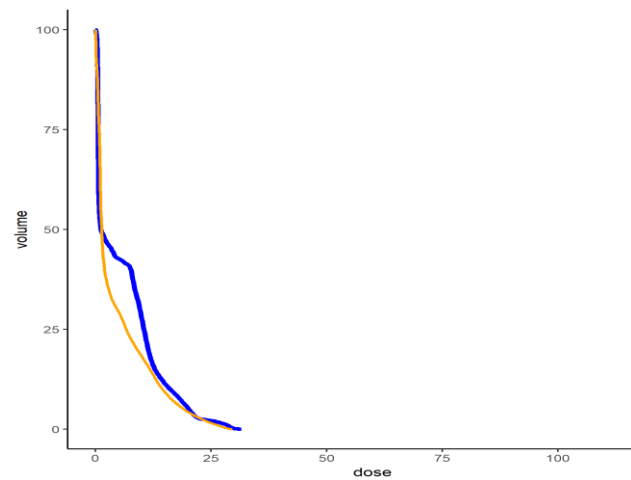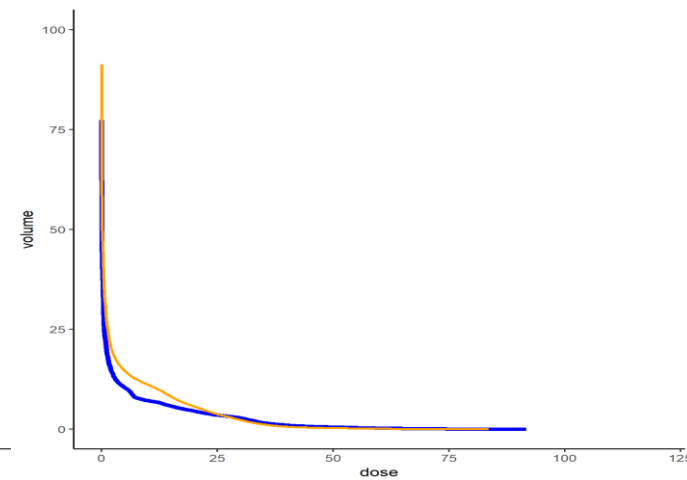

Original DVH

Predicted DVH

Case#14

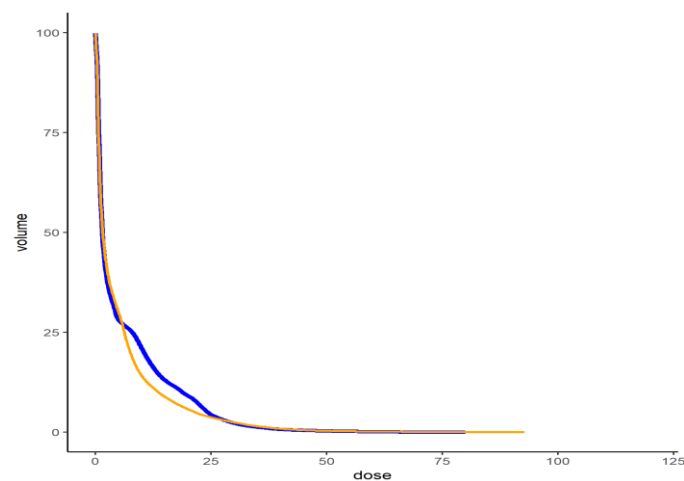

Stomach

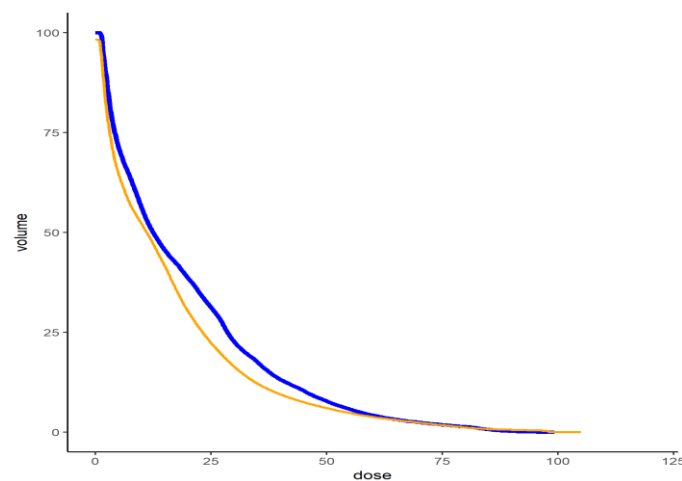

Duodenum

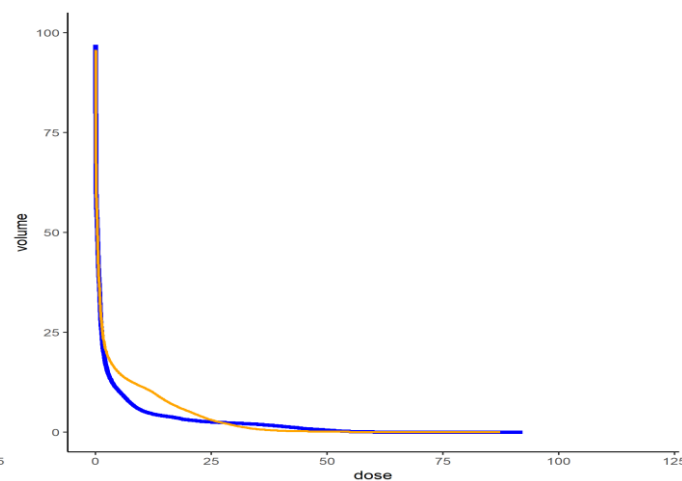

SmallBowel

Supplement: Supplementary file 5 — Fig. S5. Sample dose–volume histograms (DVHs) for three GI‐OARs from two patients (cases #11 and 14). The prediction model was generated from 10 cases (#1–10), and the DVHs in these patients are predicted. The blue line represents the actual DVH and the yellow line represents the DVHs predicted with the geometry‐driven model. [file ACM2-20-71-s005.pdf]

Sup Fig S6

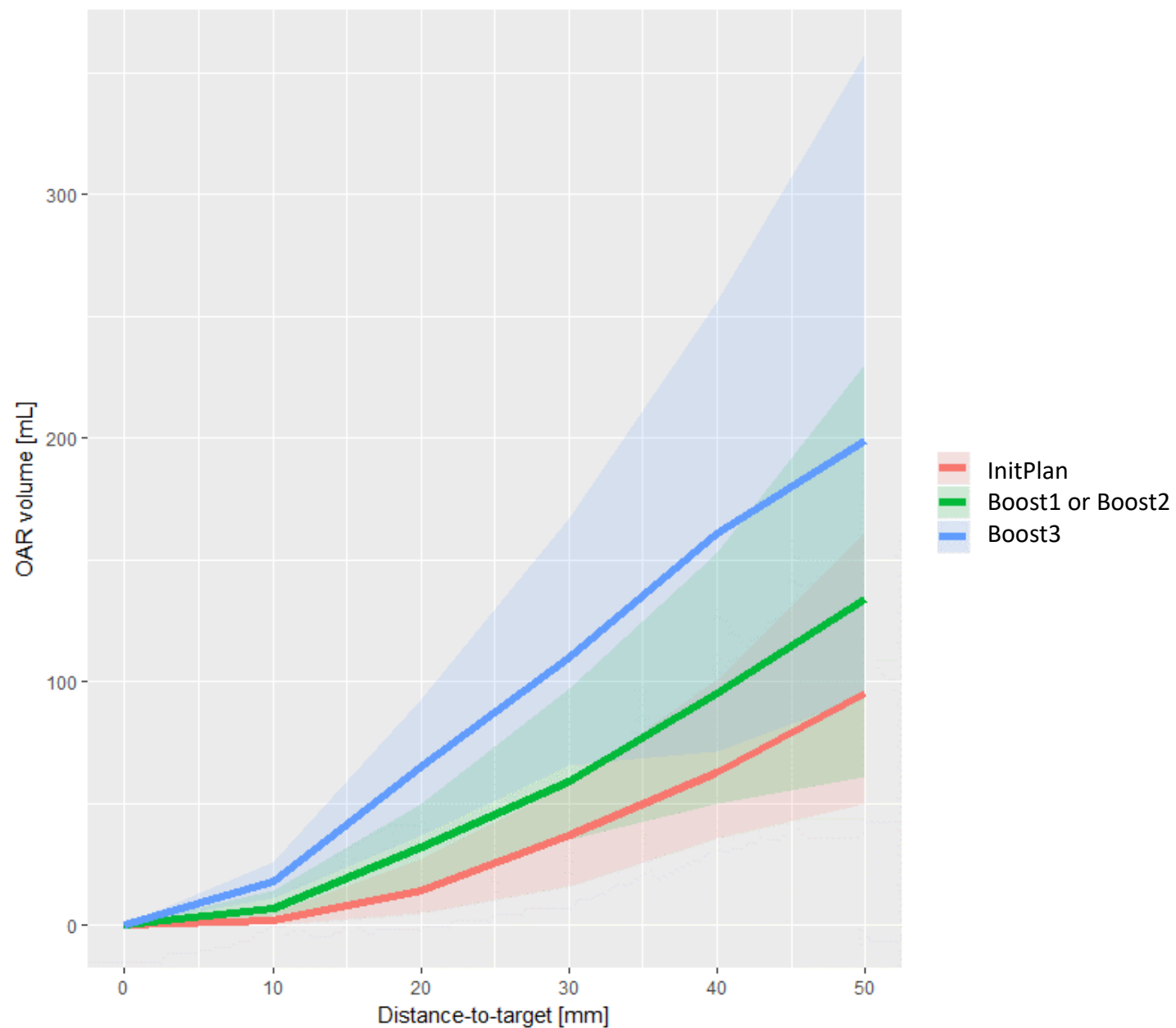

Supplement: Supplementary file 6 — Fig. S6. Overlapping volume between GI‐OARs and the expanded target volume are shown. As the size of expanded target volume (distance‐to‐target) increases, the overlapping volume increases. [file ACM2-20-71-s006.pdf]
